# Identification and mitigation of high-risk pregnancy with the Community Maternal Danger Score Mobile Application in Gboko, Nigeria

**DOI:** 10.1371/journal.pone.0275442

**Published:** 2022-09-29

**Authors:** Rajan Bola, Fanan Ujoh, Ronald Lett

**Affiliations:** 1 Canadian Network for International Surgery, Vancouver, Canada; 2 Center for Sustainability and Resilient Infrastructure and Communities, London South Bank University, London, United Kingdom; University of Mississippi Medical Center, UNITED STATES

## Abstract

**Introduction:**

Risk analyses within rural regions of Nigeria are not routinely conducted, yet could help inform access to skilled birth care. The objective of this study was to assess and compare the proportion of pregnant women at risk for maternal mortality or morbidity in Benue State, Nigeria by analysing data collected during routine antenatal visits and through the Community Maternal Danger Score (CMDS), a validated risk-analysis tool.

**Methods:**

Two cohorts, comprised of pregnant women presenting to primary healthcare centres within Gboko, Benue State between 2015–2017 and 2020–2021, were included in this study. The 2015–2017 cohort had their risk assessed retrospectively through analysis of routinely collected data. Identification of risk was based on their age, parity, and disease status (HIV and diabetes). The 2020–2021 cohort had their risk assessed prospectively using the CMDS.

**Results:**

Routinely collected data from 2015–2017 demonstrated that up to 14.9% of women in Gboko were at risk for mortality or morbidity. The CMDS reported that up to 21.5% of women were at a similar level of risk; a significant difference of 6.6% (p = 0.006). The CMDS was more efficient in obtaining and assessing this data, and the identification of risk occurred in real-time.

**Conclusion:**

Routine data collected in Gboko identifies a high proportion of pregnant women at risk for mortality or morbidity. The CMDS is an evidence-based risk analysis tool that expands on this assessment by also estimating individual and community-level risk, which allows for more efficient mitigation and prevention strategies of maternal mortality.

## Introduction

Records from the World Health Organization indicate that sub-Saharan Africa accounts for 66% of global maternal deaths, with a maternal mortality ratio of 546 per 100,000 live births [1]. The Nigerian maternal mortality ratio is 512 per 100,000 live births [2], while we report a maternal mortality ratio for Benue State, one of Nigeria’s 36 states, of 1,189 per 100,000 live births [3]. The National Demographic and Health Survey reveals that Nigeria constitutes approximately 1% of the world population yet accounts for 10% of the world maternal mortality [2]. This trend has not improved: as of 2017 a Nigerian woman’s chance of dying from pregnancy and childbirth is 1 in 13, whereas it is 1 in 5,000 in developed nations [4].

Access to skilled birth attendants (SBAs) is a necessary requirement in the care of pregnant women as it holds the potential to reduce maternal and infant mortality and morbidity rates in regions where they are employed [5]. An SBA is a health professional, such as a midwife, doctor, or nurse who has been educated and trained to manage pregnancies, childbirth, and the immediate postnatal period [6]. SBAs also monitor and counsel pregnant women on diseases such as HIV, diabetes, and syphilis [7–9]. Midwives and nurses make up the majority of SBAs [10].

For developing countries, there is a proposed target of one SBA for every 5,000 population or one SBA to attend 200 births annually [5, 11], while a lower target was set for SBAs in high-resource countries to attend between 30–120 deliveries annually [12]. Studies show that countries have successfully reduced maternal mortality rates by increasing local capacity to train, recruit, and support SBAs at deliveries [13–15].

Pregnant women are encouraged to travel to primary healthcare centres (PHCs) to deliver with an SBA, however, the geographic distribution of PHCs has a major role in access; there are inequalities in the provision of PHCs across Benue State, which may be a contributory factor impacting the decision to seek SBAs by pregnant women [16].

Analysis of routinely collected maternal data can identify pregnant women who are at risk of mortality or morbidity [3]. The Community Maternal Danger Score (CMDS) is a low-cost, evidence-based maternal risk analysis tool that was validated in Makurdi, Nigeria to predict the need for skilled birth attendance and identify high risk for maternal death [3]. The CMDS has been developed into a scoring tool that is available as an Android application to encourage pregnant women to deliver with SBAs. Pregnant women are assessed by healthcare workers in PHCs who use this scoring system at their initial visit to obtain a prenatal score out of 5 and at the 3^rd^ trimester to obtain a perinatal score also out of 5. The total score out of 10 points provides the midwives and the pregnant women with a quantitative measurement of their risk. A CMDS score of 3 out of 10 or higher is suggested to be the threshold of high risk for pregnant women [3].

To strengthen the provision of risk information, women are provided their risk score in writing through SMS messages, while also advising them on ongoing antenatal care, being proactive about danger signs of their pregnancy, and other advice on seeking SBAs at PHCs and hospitals. The CMDS is based on 7 risk factors, however due to limited capacity for obstetrical data collection in Benue State, only 3 of the 7 factors were noted to be routinely recorded in obstetrical assessments [17]. These risk factors include age, parity, and co-existing medical conditions (human immunodeficiency virus (HIV) and diabetes) [18–21].

In this study, we evaluated the proportion of pregnant women at risk for mortality or morbidity using their age, parity, and disease status in one of the most populous local government areas (LGAs) of Benue State: Gboko LGA. Our primary objective was to assess and compare the proportion of women at risk using data available from PHCs and the CMDS.

## Methods

### Study design and setting

This is a cohort study of pregnant women presenting for antenatal care at PHCs within Gboko LGA. Benue State, located in North-central Nigeria, contains 23 LGAs, including the study location of Gboko. Benue state has a population of 5.7 million, which is approximately 3% of Nigeria’s total population. Gboko accounts for 7.6% of the State’s total population of 5.7 million. Gboko is the traditional seat of authority of the *Tiv*, who are the largest ethnic group in the State. Antenatal data in Gboko was collected by randomly sampling 50% of PHCs within the LGA. The population of interest for this study is women who presented for antenatal care in Gboko between 2015–2017 and 2020–2021. No exclusion criteria were imposed.

### Data sources

Data from pregnant women over 3 years (2015–2017) were collected from medical records of 17 PHCs in Gboko in 2019. The proportion of women with risk factors was retrospectively ascertained using age, parity, and disease status (HIV or diabetes). This was considered a routine risk identification through the available maternal data. Validated criteria to define maternal risk in Benue State have not been proposed in the literature. Thus, based on criteria commonly used to assess risk by SBAs in Benue State, women from this retrospectively assessed cohort with up to 1 matching risk factor were considered low risk, women with 2 matching factors were considered high risk, and women with 3 factors were considered extremely high risk. All PHC data was categorized using definitions incorporated by the CMDS. Age was sorted into three categories: teens (ages 12–19), adults (ages 20–34), and older adults (ages 35 and older). The women were considered nulliparous if it was their first pregnancy, multiparous if pregnant between 1–4 times, and grand-multiparous if pregnant 5 or more times.

Furthermore, we prospectively applied the CMDS to a second cohort of women who presented to PHCs in Gboko between 2020–2021. Data collection included the full set of CMDS variables, and the women were scored according to the CMDS algorithm (S1 Table). Pregnant women with scores of 3 out of 10 or higher were considered high risk.

### Statistical analysis

Data analyses were based on basic descriptive statistics and Chi-Squared Test for proportions. Univariate analyses highlighting proportions and counts were calculated for age, disease status, and parity of all pregnant women. The proportion of women at risk for mortality or morbidity based on their age, parity, and disease status was estimated for the cohort of women from 2015–2017. We describe the median CMDS score, report interquartile ranges, and estimate the proportion of women at risk for the 2020–2021 cohort. We compared the proportions of women at risk for mortality or morbidity between the two cohorts from 2015–2017 and 2020–2021 using Chi-Squared Test. Statistical significance was assumed at a p-value less than or equal to 0.05. R 4.1 was used for statistical analysis (Vienna, Austria).

### Ethical considerations

Approval for the study team, comprised of local and international researchers, to collect antenatal data was obtained from the Benue State Ministry of Health and Human Services, Makurdi. Part of the conditions listed in the ethical approval was to anonymize the patients and that data may only be shared with external researchers upon formal request.

## Results

The data collected from the retrospective cohort came from 17 PHCs and include a total of 604 patients (Table 1). Teens accounted for 14.2% of the sample, adults comprised 79.8%, while older adults made up 6.0%. Nulliparous women comprised 9.3% of the sample, multiparous women accounted for 81.4%, and grand-multiparous women accounted for 9.3%. 256 women had either diabetes or HIV, while 1 woman had both. 347 women did not have either HIV or diabetes.

**Table 1:** Counts and proportions of pregnant women in Gboko based on age, parity, and disease status measurements by cohort year.

Condition and Category		2015–2017		2020–2021		P-value
		*Count*	*Proportion*	*Count*	*Proportion*	
*Age*	Teenager	n = 86	14.2%	n = 72	16.6%	p = 0.6773
	Adult	n = 482	79.8%	n = 320	73.9%	p = 0.0504
	Older Adult	n = 36	6.0%	n = 41	9.5%	p = 0.5717
*Parity*	Nulliparous	n = 56	9.3%	n = 196	45.3%	p<0.001
	Multiparous	n = 492	81.4%	n = 176	40.6%	p<0.001
	Grand-Multiparous	n = 56	9.3%	n = 61	14.1%	p = 0.4235
*Disease Status*	No Diseases	n = 347	57.5%	n = 430	99.3%	p<0.001
	HIV or Diabetes	n = 256	42.3%	n = 3	0.7%	p = 0.1472
	HIV and Diabetes	n = 1	0.2%	n = 0	0%	N/A

The data collected prospectively between 2020–2021 include 433 patients (Table 1). Teens comprised 16.6% of the cohort, adults 73.9%, and older adults 9.5%. Nulliparous women accounted for 45.3% of the sample, while multiparous women accounted for 40.6%, and grand-multiparous accounted for 14.1%. Only 3 women were reported to have either diabetes or HIV.

The two cohorts differed in the proportion of nulliparous (p<0.001) and multiparous women (p<0.001), as well as the number of women with no diseases (p<0.001).

### Routine risk identification and CMDS comparison

Of the 604 women from the retrospective cohort, 212 women were not identified to be at risk based on the factors examined. Up to 85.1% of women (n = 302) were at low risk for maternal mortality or morbidity, 13.2% (n = 80) were at high-risk for mortality or morbidity, and 1.7% (n = 10) were at extremely high risk for mortality or morbidity. At least 14.9% of women from the retrospective cohort had a high level of risk and required an SBA at delivery.

**Fig 1:**
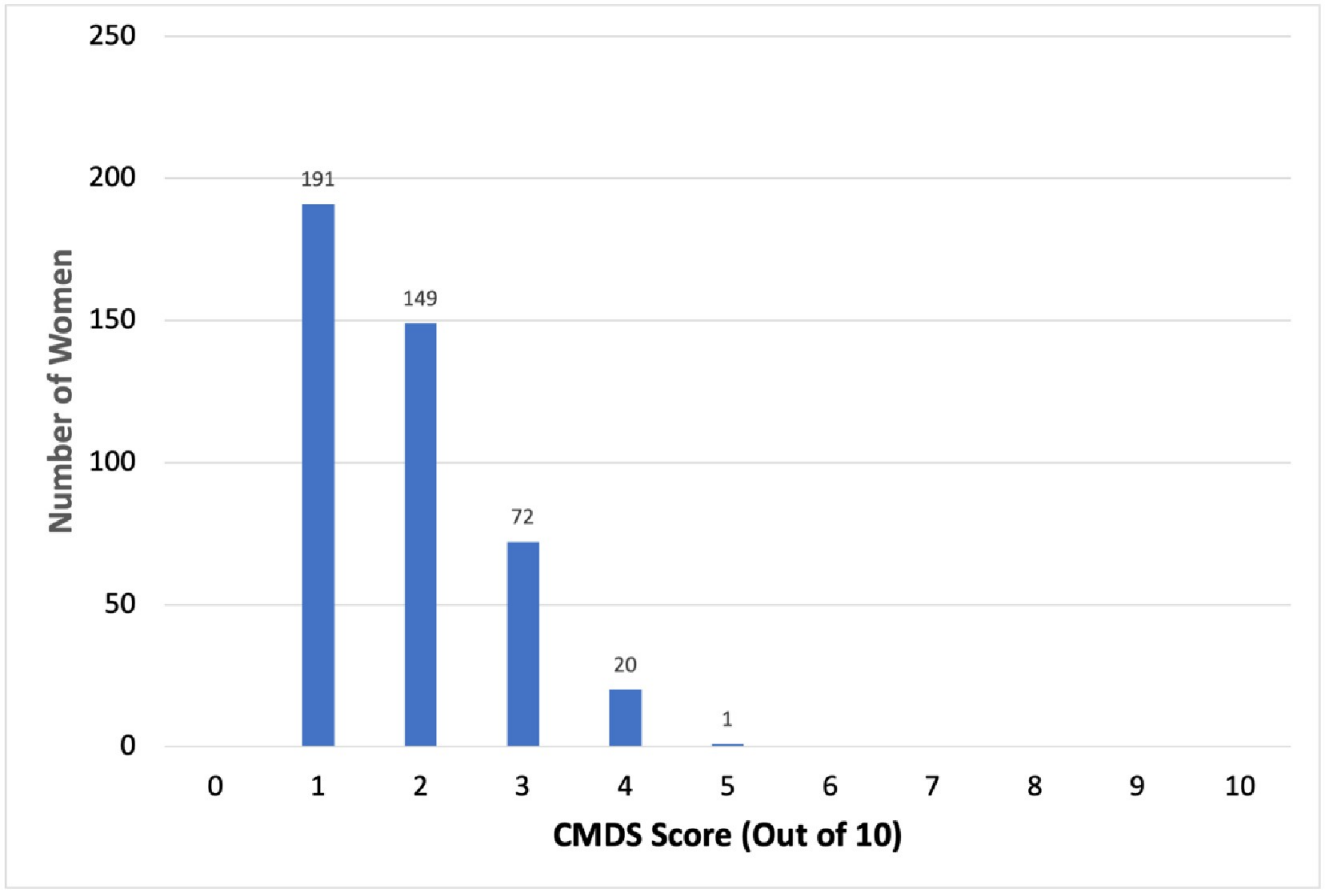
Distribution of CMDS scores for a cohort of pregnant women in Gboko, 2020–2021.

The women who were scored prospectively using the CMDS guidelines had a median score of 2 out of 10 with an interquartile range between 1 and 3. None of the women were scored as 0 out of 10. 191 women (44.1%) were scored as 1, 149 women (34.5%) were scored as 2, 72 women (16.6%) were scored as 3, 20 women (4.6%) were scored as 4, and 1 woman (0.2%) was scored as 5. The CMDS score distribution for this cohort of women is shown in Fig 1. Based on the CMDS threshold of risk of 3 points or higher, 21.5% of women from the prospective cohort were considered high-risk and provided SMS text messages to encourage them to seek an SBA in preparation for delivery.

Based on the routine risk identification for the 2015–2017 cohort of women, which identified 14.9% of women at risk, and CMDS scoring for the 2020–2021 cohort of women, which identified 21.5% of women at risk, there was a significant difference of 6.6% of women identified at risk between these cohorts (p = 0.006). The CMDS was not only able to identify more women at high risk than through the analysis of routinely collected data, but it was able to do so in real time.

## Discussion

In this study, we report an estimate of the proportion of women at risk for maternal mortality or morbidity in Benue State of 21.5%. The inclusion of two temporally distinct cohorts in this study allowed for the determination of maternal characteristics of pregnant women in Gboko. It was found that the baseline characteristics of the population had not considerably changed in the prior 4 years, however there were differences in the proportions of women seeking skilled birth care based on their parity and disease status.

The routine risk analysis in Gboko demonstrated that between 2015–2017, approximately 15% of women needed an SBA in preparation for a safe delivery. This result is the first analysis of risk in Benue State depicting a large proportion of pregnant women who require an SBA at delivery. These findings demonstrate that there is a considerable proportion of pregnant women at risk within the community, yet local healthcare teams and policy makers do not routinely collect this maternal data to assess baseline risk in the community. This is likely due to the inefficiencies involved in this process, as well as lag-time between data collection, analysis, and dissemination which makes large-scale data collection impractical.

The CMDS was used to prospectively score the cohort of pregnant women from 2020–2021 to ascertain the level of risk using a more comprehensive assessment developed from evidence-based maternal risk factors. In addition to reporting a significantly higher proportion of women at risk of 21.5% (p = 0.006), the CMDS was able to identify this level of risk in real-time, and subsequently recommend prevention strategies to at-risk women in an efficient manner.

The results obtained for the proportions of at-risk pregnant women were comparable to findings from hospitals in Malaysia and Nepal, which reported proportions of 11% and 15%, of women at risk, respectively [22, 23]. However, our findings differed from outpatient clinics in India and Malaysia, which reported proportions of women at-risk upwards of 31% [24, 25]. A notable difference between these studies was the method used to define the proportion of pregnant women at risk; the criteria were often based on healthcare provider intuition and may not have been validated among different populations and settings.

Benue State has the potential to decrease maternal mortality by identifying women at high risk for maternal mortality through applications like the CMDS. The CMDS was designed to improve the maternal health outcomes of women in low-resource settings where access to skilled care can be limited and where obstetrical data is only available years later and inaccessible to the women cared for. The CMDS was shown to identify women at risk for mortality in a manner that was more efficient than conventional data management methods at PHCs. The CMDS provided comprehensive assessment on an expanded set of variables to standardize and improve on data collected at PHCs, while also incorporating best-practice guidelines. Integrating the CMDS in routine data collection efforts would prove to be an effective strategy for collecting maternal data across rural regions. This data can then be used to inform health resources planning, epidemiological evaluations, and risk assessments within Gboko, and possibly other regions impacted by high maternal mortality rates.

### Limitations

The primary limitations of this study were that the two cohorts were limited in sample size which affected the number of included women in extremely high risk categories. Both cohorts only included data from women who were seeking care at PHCs and may not be generalizable to pregnant women who are unable to seek antenatal care. Including a larger sample of women from community settings who were unable to seek antenatal care would possibly address these limitations and promote generalizability of results.

## Conclusion

We determine that the current antenatal data collection efforts at primary healthcare centres are ineffective at supporting community-level risk assessments within the State due to the limited number of variables that are routinely collected. Mobile health technologies like the Community Maternal Danger Score can efficiently standardize data collection and inform healthcare planning and resource allocation, ultimately to improve maternal care. Future studies are required to assess the direct impact on care-seeking behaviours of pregnant women and improvements to maternal outcomes that the Community Maternal Danger Score has in low-resource settings.

## Supporting information

S1 Table Scoring criteria of the CMDS for women in Gboko (2020–2021). (DOCX)
