# Supplementary material for: Identification and mitigation of high-risk pregnancy with the Community Maternal Danger Score Mobile Application in Gboko, Nigeria
Source: PLoS One. 2022 Sep 29;17(9):e0275442. doi: 10.1371/journal.pone.0275442 (PMC9521834; doi:10.1371/journal.pone.0275442)
Supplement: S1 Table — (DOCX) [file pone.0275442.s001.docx]

**Supplement Table 1. Scoring Criteria of the CMDS for Women in Gboko (2020-2021).**

| **Name of Risk Factor** | **Requirement for 2 Points** | **Requirement for 1 Point** | **Requirement for 0 Points** |
| --- | --- | --- | --- |
| Age | - N/A | - Below 20 years of age - 35 years of age or older | - 20 years of age or older and below 35 years of age |
| Parity | - N/A | - Nulliparous (para = 1) - Grand-multiparous (para > 4) | - Multiparous (para 1-4) |
| Patient Size | - N/A | - Underweight (BMI ≤ 18.5 kg/m^2^) - Overweight (BMI > 30.0 kg/m^2^) | - Normal weight (BMI > 18.5 and ≤ 30.0 kg/m^2^) |
| Obstetrical History | Two of the following:   - Previous hemorrhage - Previous stillbirth or miscarriage - Previous breech delivery - Previous twins (or more) - Previous pregnancy within 1.5 years or greater than 5 years ago - Reported reduction in fetal movements | One of the following:   - Previous hemorrhage - Previous stillbirth or miscarriage - Previous breech delivery - Previous twins (or more) - Previous pregnancy within 1.5 years or greater than 5 years ago - Reported reduction in fetal movements | - Lack of these conditions |
| Fundal Height (3^rd^ trimester) | - N/A | - Fundal height ≤35cm - Fundal height >40cm | - Fundal height >35cm and ≤40cm |
| Signs of Pre-Eclampsia | - Very High Blood pressure (≥140 systolic and ≥100 diastolic mmHg)   OR   - Any sign of pre-eclampsia: proteinuria, headache, epigastric pain, blurred vision, excessive weight gain of 1kg or greater per week, or seizures   AND   - High blood pressure (>120 systolic or >90 diastolic mmHg) | - High blood pressure (>120 systolic or >90 diastolic mmHg) | - Normal blood pressure (≤120 systolic and ≤90 diastolic mmHg) - Lack of the aforementioned signs |
| Co-existing Conditions | Two of the following:   - HIV - Anemia - Tuberculosis - Female genital mutilation - Diabetes - Malaria   OR  One of the following:   - Maternal sepsis - Fever with ruptured membranes | One of the following:   - HIV - Anemia - Tuberculosis - Female genital mutilation - Diabetes - Malaria | - Lack of the aforementioned conditions |
